# *Nigella sativa* Fixed and Essential Oil Supplementation Modulates Hyperglycemia and Allied Complications in Streptozotocin-Induced Diabetes Mellitus

**DOI:** 10.1155/2014/826380

**Published:** 2014-01-08

**Authors:** Muhammad Tauseef Sultan, Masood Sadiq Butt, Roselina Karim, M. Zia-Ul-Haq, Rizwana Batool, Shakeel Ahmad, Luigi Aliberti, Vincenzo De Feo

**Affiliations:** ^1^Faculty of Food Science and Technology, University of Putra Malaysia (UPM), Malaysia; ^2^National Institute of Food Science & Technology, University of Agriculture, Faisalabad, Pakistan; ^3^The Patent Office, Karachi, Pakistan; ^4^Department of Agronomy, Bahauddin Zakariya University, Multan 60800, Pakistan; ^5^Department of Pharmacy, University of Salerno, Via Giovanni Paolo II 132, Fisciano, 84084 Salerno, Italy

## Abstract

In the recent era, diabetes mellitus has emerged as one of the significant threats to public health and this situation demands the attention of the researchers and allied stakeholders. Dietary regimens using functional and nutraceutical foods are gaining wide range of acceptance and some traditional medicinal plants are of considerable importance. The main objective of this instant study was to explore the antidiabetic potential of *Nigella sativa* fixed oil (NSFO) and essential oil (NSEO). Three experimental groups of rats received diets during the entire study duration, that is, D_1_ (control), D_2_ (NSFO: 4.0%), and D_3_ (NSEO: 0.30%). Experimental diets (NSFO & NSEO) modulated the lipid profile, while decreasing the antioxidant damage. However, production of free radicals, that is, MDA, and conjugated dienes increased by 59.00 and 33.63%, respectively, in control. On the contrary, NSFO and NSEO reduced the MDA levels by 11.54 and 26.86% and the conjugated dienes levels by 32.53 and 38.39%, respectively. *N. sativa* oils improved the health and showed some promising anti-diabetic results.

## 1. Introduction

In the domain of diet-based therapies, functional foods are important to combat lifestyle related disorders, that is, hyperglycemia, high cholesterol, and immune dysfunction. Such functional foods, nutraceuticals, and pharma foods are modern trends [1] and utilization of medicinal plants is gaining wide range of recognition [2, 3]. Various plants rich in functional ingredients possess the ability to reduce hyperglycemia and hypercholesterolemia along with quenching free radicals [4]. The bioactive molecules present in them, including antioxidants, phytosterols, and flavonoids, are responsible for health claims associated with the plants. Recent research studies also validated some traditional health claims of certain plants and whole scenario led dietetics/nutritionists to consider them suitable in diet-based medication of various illnesses [5].

Diabetes mellitus is one of the leading causes of mortality all over the globe and targets multiorgan systems [6]. According to World Health Organization estimates, more than 376 million people will be diabetic globally in 2030 and about two billion people would be at risk due to poor dietary habits, obesity, and lack of physical exercise [7]. It is worth mentioning that healthy lifestyle and dietary measures can prevent 30–40% of all kinds of diseases. Diet diversification or slight changes in the daily diet can possibly prevent the onset of diabetes mellitus [8]. Therefore, the utilization of natural foods rich in bioactive compounds/functional ingredients is gaining wide range of acceptance. These natural compounds act as micronutrients and there are growing efforts in exploring molecular basis for their therapeutic mechanisms [4]. However, diet selection is imperative for the management of diabetes and its allied complications. Scientists over the globe believe that dietary modifications are important along with pharmaceuticals for the treatment of diabetes mellitus and allied complications [9].

In the last few decades, scientists explored many plants possessing antidiabetic perspectives, for example, garlic, bitter melon, green tea, fenugreek, pelargonium, turmeric, rice bran, oat, mulberry, amaltas, and so forth, [10]. Researchers over the globe have recently focused their studies on the possible role of *Nigella sativa *L. (*Ranunculaceae*) or black cumin for management of diabetes. Extracts of *Nigella sativa* (also known as black cumin or black seeds) possessed blood glucose lowering effects, but the exact anti-diabetic mechanism is not yet established. Hypoglycemic effects of black cumin oil might be due to presence of some phytochemicals including thymoquinone and carvacrol [11]. *N. sativa* fixed oil is rich in polyunsaturated fatty acids and some minor dihomolinolenic acids, tocopherols, and phytosterols. In comparison, *N. sativa* essential oil is rich source of antioxidants including thymoquinone, *p*-cymene, carvacrol, anethole, and 4-terpineol [12, 13]. These fractions of *N. sativa* might be helpful in lowering hyperglycemia through *β*-cell integrity and enhancing the insulin secretions. Moreover, presence of functional ingredients and bioactive molecules could provide protection against diabetes complication [14].

In the present study, we attempted to explore *N. sativa* fixed (NSFO) and essential oils (NSEO) for their antidiabetic properties, through the estimation of values of blood glucose, insulin, serum lipid profile, and indices of oxidative damage.

## 2. Materials and Methods

### 2.1. Plant Material

The Barani Agricultural Research Institute, Chakwal, provided *N. sativa* seeds. A voucher specimen (Voucher/Specimen no. Chk. Pk-926) of the plant is preserved in the herbarium of the same Institute.

### 2.2. Chemicals

Chemical reagents (analytical and HPLC grade) like xylenol orange (o-cresosulfonphthalein-3,3-bis (sodium methyl iminodiacetate)) and standards were purchased from Sigma-Aldrich Tokyo, Japan, and Merck KGaA, Darmstadt, Germany.

### 2.3. Extraction of *Nigella sativa* Fixed and Essential Oils

Following the standard procedures, the seeds of *N. sativa* were slurred with hexane (in the ratio of 1 : 6 using a Soxlet apparatus and rotary evaporator was used to remove solvent) to extract the fixed oil. In contrast, the essential oil was extracted using locally assembled hydrodistillation apparatus.

### 2.4. Animals

The National Institute of Health (NIH), Islamabad, provided infectious free 30 Sprague Dawley rats that were further divided into three groups of ten rats each. The animals were maintained according to standard guidelines of Animal Institute of Nutrition (AIN), USA, that is, temperature 23 ± 2°C, relative humidity 55 ± 5%, and 12-hr light-dark cycle. In the first week, the feed of the rats was a basal diet in order to acclimatize them to new environment. Later, rats received their respective experimental diets for a period of eight weeks (56 days) as reported in Table 1.

The analytical procedures carried out include parameters measured daily (feed and water intake) and body weight (weekly basis). At 28 and 56 days of feeding trials, five rats from each group were decapitated to collect blood through cardiac and neck puncture [15].

### 2.5. Induction of Diabetes Mellitus

Diabetic mellitus was induced in rats (weight 150–200 g) by injecting intravenously streptozotocin (STZ) in a dose of 60 mg/Kg body weight, dissolved in 0.01 M citrate buffer (pH 4.5). The blood glucose level of each rat was monitored after injecting STZ to check the glucose response. The rats received experimental diets after the mean values for blood glucose reached >200 mg/dL.

### 2.6. Blood Glucose and Insulin Levels

In order to check the hypoglycemic effects of NSFO and NSEO, we determined the glucose concentration of individual rat in each study using GOD-PAP method as described by [16], while insulin level was determined following the method of Besch et al. [17].

### 2.7. Blood Lipid Profile

The blood samples from each group of rats were centrifuged at 3000 rpm and the serum was collected as supernatant layer. The collected serum was used for the estimation of serum lipid profile and cholesterol by CHOD-PAP method [18], high density lipoprotein (HDL) by HDL cholesterol precipitant method [19], total triglycerides by liquid triglycerides (GPO-PAP) method [20], and low density lipoproteins (LDL) following the procedure of McNamara et al. [21]. For estimations of the aforementioned parameters, the standard procedures mentioned on the commercial kits without any further modifications were used. Briefly, serum cholesterol and triglyceride levels were measured by using enzymatic reagents adapted to a MicroLab-300 (Merck, Germany). Similarly, phosphotungstic acid and magnesium chloride based assay was used to estimate high-density lipoprotein (HDL).

### 2.8. Indices of Oxidative Damage

Indicators of lipid peroxidation were estimated including MDA level [22], total antioxidant capacity using xylenol orange assay [23], and conjugated dienes according to protocol described by Corongiu and Milla, [24]. For total antioxidant capacity, reagent-1 (containing xylenol orange, NaCl, and glycerol) was added to 35 *μ*L of collected serum and then to 11 *μ*L of reagent-2 (containing ferrous ion and *o*-dianisidine in H_2_SO_4_ solution). The readings were taken at 560 nm and 800 nm at the start of the reaction and at the end of the reaction (3-4 min). Briefly, we measured MDA with a colorimetric method using tetraethoxypropane as standard in nmol/mL. The detection limit was 0.02 mmol/L and the intra- and interassay coefficients of variation were 6.1% and 7.3%, respectively. For the determination of conjugated dienes, serum was homogenized and centrifuged at 3000 g. Then the supernatant or organic layer was transferred in screw-capped micro test tubes and flushed with nitrogen gas for semidrying. The spectra obtained at wavelength of 232–247 nm using hexane as standard solution were further used to estimate conjugated dienes and values were expressed in nmol/g of lipids.

### 2.9. Statistical Analysis

Statistical package, that is, Cohort V-6.1 (Co-Stat Statistical Software, 2003), was used for data analysis. Briefly, values presented in tables are means ± standard deviation. In order to check the level of significance, analysis of variance (ANOVA) technique was applied. The diets (factor A), intervals (factor B), and their interaction (A × B) were used as source of variations. Duncan's multiple range test (DMRt) further clarified the effects of diets in a comprehensive manner.

### 2.10. Ethics

The experiments were carried out following the instructions of “Animal Care Committee, NIFSAT-Faisalabad, Pakistan”.

## 3. Results

**Figure 1:**
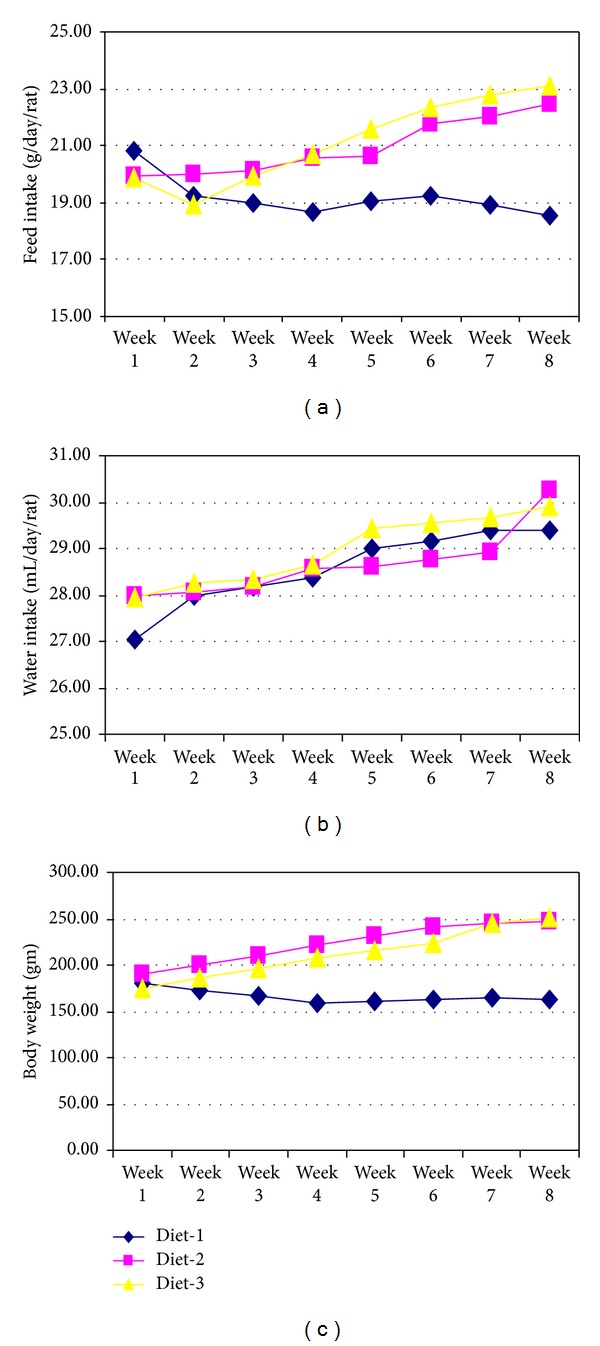
Feed and water intake and body weight of diabetic rats. D_1_ (control), D_2_ (NSFO), and D_3_ (NSEO).

The injection of 60 mg/Kg body weight streptozotocin resulted in onset of diabetic mellitus, due to damage to pancreas thus reducing the concentrations of insulin. During the course of study, feed intake (Figure 1) increased significantly (*P* < 0.05) in the experimental diets, that is, D_2_ (NSFO) and D_3_ (NSEO). In contrast, feed intake decreased significantly in D_1_ (control) group. However, water intake increased non-significantly with the passage of study from 27.65 ± 0.53 to 29.86 ± 0.43 mL/rat/day. Body weight varied significantly (*P* < 0.01) as a function of diets; maximum body weight 223.59 ± 21.49 g was observed in D_2_ (NSFO) followed by 212.49 ± 26.83 g in D_3_ (NSEO), while minimum 165.99 ± 7.03 g was recorded for D_1_ (control).

### 3.1. Hypoglycemic Potential

Blood glucose and insulin levels varied significantly (*P* < 0.05) due to diets, study intervals, and interaction. The maximum glucose level was observed in D_1_ (control) followed by D_2_ (NSFO), while minimum level was recorded in D_3_ (NSEO) treatment. During the course of the eight-week study, D_3_ group exhibited pronounced decrease in glucose from 210.47 ± 8.01 mg/dL to 188.94 ± 8.39 mg/dL (Table 2). Likewise, glucose decreased from 217.41 ± 9.09 to 208.50 ± 9.18 mg/dL in D_2_ group, whereas D_1_ group showed significant increase in glucose from baseline value of 211.78 ± 12.22 to 282.10 ± 8.20 mg/dL at the end of study. Insulin levels differed due to the diets; the diet D_3_ showed the maximum insulin concentrations followed by D_2_, while D_1_ group recorded the least insulin concentrations. Insulin level improved in fixed and essential oils group from from 40.35 ± 1.03 to 42.62 ± 0.89 and 42.38 ± 0.52 to 46.80 ± 2.08 *μ*U/mL, respectively, as compared to the substantial decrease in D_1_ group from 42.11 ± 1.35 to 20.33 ± 0.59 *μ*U/mL during the 56-day study.

### 3.2. Serum Lipid Profiles

It is obvious from statistical analysis that cholesterol and LDL differed significantly (*P* < 0.05) with diets, study intervals, and interaction. Likewise, diets affected triglycerides and HDL momentously. Means for cholesterol contents (Table 3) indicated significant variations due to diets and maximum cholesterol was recorded in D_1_ (control), while D_2_ (NSFO) and D_3_ (NSEO) groups had lower values. During the eight-week trial, in D_1_ (control) cholesterol contents increased from 106.56 ± 3.42 to 147.76 ± 4.30 mg/dL. Experimental diets containing oils of *N. sativa *fixed and essential oils decreased the same trait significantly (*P* < 0.05), with more pronounced effects in D_2_ from 110.36 ± 2.81 to 98.97 ± 2.08 mg/dL, whereas D_3_ decreased that said trait from 116.31 ± 1.42 to 105.97 ± 4.70 mg/dL. The D_1_ diet showed the maximum LDL contents (67.97 ± 10.58 mg/dL), while minimum contents 46.22 ± 3.69 mg/dL recorded in D_2_ group. The results regarding HDL indicated that the maximum HDL, 41.86 ± 0.71 and 40.90 ± 0.91 mg/dL was recorded in D_2_ and D_3_ groups, respectively, as compared to minimum 37.52 ± 0.46 mg/dL in D_1_. Similarly, maximum triglycerides 112.25 ± 9.06 mg/dL were recorded in groups of rats fed on D_1_ followed by D_2_ and D_3_ with mean triglycerides of 89.96 ± 2.15 and 95.75 ± 1.62 mg/dL, respectively.

### 3.3. Indices of Oxidative Damage

Indicators of oxidative damage like total antioxidant capacity (TAC), serum MDA, and conjugated dienes levels exhibited significant differences due to diets (*P* < 0.01) and interaction (*P* < 0.05). However, study interval remained nonsignificant except for TAC (Table 4). It is evident that during the 56-day trial, D_2_ (NSFO) and D_3_ (NSEO) improved the TAC of the serum from 0.56 ± 0.02 to 0.78 ± 0.03 IU/mL and from 0.47 ± 0.02 to 0.91 ± 0.04 IU/mL, respectively. However, total antioxidant capacity decreased momentously in D_1_ (control) from 0.52 ± 0.030 to 0.29 ± 0.02 IU/mL. Maximum MDA level (8.71 ± 1.136 nmol/g) was recorded in D_1_, whereas the minimum MDA level was noted in D_3_ group (4.67 ± 1.202 nmol/g). Conjugated dienes varied significantly (*P* < 0.05); however, maximum values were observed in D_1_ (control) groups followed by D_2_ (NSFO), and least conjugated dienes were observed in D_3_ (NSEO).

### 3.4. Correlation Matrix

The association of various parameters has been evaluated using correction matrix. This was evaluated by using the technique of multiple regression or order to check the interdependence of various variables on each other (Table 5). It is obvious from the correlation coefficients that serum glucose levels are positively associated with lipid profile, that is, cholesterol, LDL, and triglycerides (*P* < 0.01) and indices of oxidative damage, that is, MDA and conjugated dienes (*P* < 0.01); However, glucose is in negative association with insulin (*P* < 0.01) and total antioxidant capacity (*P* < 0.05). Insulin level was also found to be inversely associated with lipid profile, however, the same parameter was in linear association with that of total antioxidant capacity (*P* < 0.01). Cholesterol, LDL, and triglycerides were positively correlated with each other (*P* < 0.01) but were found to be inversely associated with HDL (*P* < 0.01) in diabetic rats modeling.

## 4. Discussion

Diabetes mellitus is one of the most common noncommunicable diseases that targets multiorgan systems. In the recent years, scientists over the globe directed their efforts for the exploration of some novel food sources as hypoglycemic agents [6]. In the early stages, drugs and diet can mediate the adverse consequences. If there arose complications like cardiovascular disorders or renal malfunction, the strategies need to focus through some alternative arrangements. However, prevention of root causes and managing diabetes at early stages is the better remedy. Under such circumstances, natural therapies including the use of medicinal plants, functional foods, and nutraceuticals are important [5, 25].

Many researches have studied the possible role of *Nigella sativa* fixed and essential oils for the management of diabetes. In the present investigation, *N. sativa* fixed and essential oils decreased the blood glucose significantly. The group of rats fed on control diet witnessed the abrupt increase by twofold in blood glucose during the 56 days of study duration. Diets containing NSFO and NSEO resulted in enhanced insulin secretions as compared to substantial decrease in control group. The increased insulin secretion certainly is a possible reason for the drop in glucose level in experimental diets groups. The present statistics proved that *N. sativa* fixed and essential oils hold insulinotropic potential and mediated by extra pancreatic action. Supportive evidences were presented by Kanter [26], who observed that *N. sativa* and its constituent thymoquinone, at a dose of 400 and 50 mg/kg body weight/day, caused a marked decrease in glucose and increased the serum insulin concentrations in streptozotocin-induced diabetic rats. Earlier, Meral et al. [27] observed lower glucose levels in diabetic rats treated with *N. sativa* in comparison to control. Moreover, hepatic glucose production contributes significantly to hyperglycemia in diabetic patients. It tends to decrease, due to *N. sativa *treatments as reported by Fararh et al. [28]. The aforementioned reports suggested that the mechanisms behind hypoglycemic potential could include increased insulin concentrations, protection of the *β*-cells of islets of Langerhans, prevention of oxidative damage, and extra pancreatic action [11].

Diabetes mellitus results in frequent changes in the plasma lipid concentration that certainly contribute to the development of vascular diseases [1]. In the present investigation, cholesterol, triglycerides, and LDL increased in the control group during the entire study. In contrary, NSFO treatment reduced the same traits by 10.32, 7.89 and 23.74%, respectively, whilst NSEO treatment decreased the 8.89%, 5.33, and 19.89% cholesterol, triglycerides, and LDL, respectively. Lipid lowering potential is substantially beneficial for the subjects with diabetes and multiple cardiovascular risk factors. Kanter et al. [11] and Meral et al. [27] hypothesized that the cholesterol deposition is due to increased activities of cholesterol synthesizing enzyme and reduced activities of antioxidants. Considering the fact that the serum cholesterol was in negative association with tocopherols, the supplementation of diets with NSFO (rich source of tocopherols) and NSEO (rich source of antioxidant) can reduce the elevated cholesterol level [29].

*N. sativa* inhibited the lipid peroxidation of biological membranes and prevented the lipid-peroxidation-induced liver damage in diabetic rabbits [27]. The results of El-Missiry and El-Gindy [30] showed marked increase in lipid peroxides and decreased antioxidant enzymes in diabetes mellitus. Later, Türkdoĝan et al. [31] observed occurrence of oxidative decomposition of liver because of increased lipid peroxidation [27]. This effect might be due to increased level of antioxidants production that protects tissues from the hazards of free radicals. The bioactive molecules present in such functional foods, for example, *α*-lipoic acid, tocopherols, and selenium, are helpful to control diabetes and its complications. In similar type of rats modeling, Fararh et al. [28] enumerated the differences in feed intake and body weight of diabetic rats. *N. sativa* essential oil might mediate hypoglycemic impact through extra pancreatic actions, stimulated insulin release, and partial regeneration/proliferation of pancreatic *β* cells [11].

There are several evidences that complications related to diabetes are associated with oxidative stress, induced by reactive oxygen species. Accordingly, interest has grown in using natural antioxidants for prevention or protection against oxidative damage [14]. The results of the present exploration indicated that production of free radicals, that is, MDA and conjugated dienes, increased by 59.00 and 33.63%, respectively, in control, whilst groups of rats fed on *N. sativa* fixed and essential oils based diets improved the antioxidant defense mechanism by reducing the MDA and conjugated dienes levels. In this study, hyperglycemia was linearly associated with antioxidant damage, that is, MDA and conjugated dienes levels, whilst it was inversely correlated with total antioxidant capacity and glutathione contents. STZ results in depletion of antioxidant system in both blood and tissues and promotes the generation of free radicals [32]. *N. sativa* essential oil owing to its antioxidant potential is useful in controlling the diabetic complications in experimental diabetic rats. Results of present study supported the traditional use of *N. sativa* and its derived products as a treatment for hyperglycemia and related abnormalities. Moreover, *Nigella sativa* fixed and essential oils significantly ameliorate free radicals and improve antioxidant capacity, thus reducing the risk of diabetic complications.

## 5. Conclusion

The experimental diets containing *Nigella sativa* fixed and essential oil possess hypoglycemic properties; NSEO was more effective in reducing the extent of oxidative damage. Moreover, both extracts improved significantly the lipid profile including total cholesterol, triglycerides, and LDL and reduced the serum malonaldehyde level, improving antioxidant capacity of the body. Data obtained in the present study are helpful in designing further studies in human subjects in order to validate the use of *N. sativa *oils in prevention and in treatment of diabetes and related conditions.

## Figures and Tables

**Table 1:** Diet plan used in the study.

	Diets
Group-I	D_1_: (control/placebo diet)
Group-II	D_2_: (4.0% fixed oil)
Group-III	D_3_: (0.3% essential oil)

**Table 2:** Effects of diets containing NSFO and NSEO on blood glucose and insulin levels in diabetic rats.

Parameters	Diets	Study intervals (days)		
		0	28	56
Glucose (mg/dL)	D_1_ (control)	211.78 ± 12.22^c^	241.67 ± 7.83^b^	282.10 ± 8.20^a^
	D_2_ (NSFO)	217.41 ± 9.09^c^	214.28 ± 7.20^c^	208.50 ± 9.18^c^
	D_3_ (NSEO)	210.47 ± 8.01^c^	195.36 ± 9.51^d^	188.94 ± 8.39^e^

Insulin (*μ*U/mL)	D_1_ (control)	42.11 ± 1.35^b^	32.07 ± 1.04^d^	20.33 ± 0.59^e^
	D_2_ (NSFO)	40.35 ± 1.03^bc^	38.68 ± 0.70^c^	42.62 ± 0.89^b^
	D_3_ (NSEO)	42.38 ± 0.52^b^	45.44 ± 1.34^a^	46.80 ± 2.08^a^

Means sharing same letters in a column/row do not differ significantly at *P* < 0.05.

**Table 3:** Effects of diets containing NSFO and NSEO on cholesterol, HDL, LDL, and triglycerides levels in diabetic rats.

Parameters	Diets	Study intervals (days)		
		0	28	56
Cholesterol (mg/dL)	D_1_ (control)	106.56 ± 3.42^de^	130.37 ± 4.22^b^	147.76 ± 4.30^a^
	D_2_ (NSFO)	110.36 ± 2.81^cd^	108.90 ± 1.98^cd^	98.97 ± 2.08^e^
	D_3_ (NSEO)	116.31 ± 1.42^c^	113.92 ± 3.36^c^	105.97 ± 4.70^de^

HDL (mg/dL)	D_1_ (control)	38.36 ± 2.21	36.79 ± 1.19	37.40 ± 1.90
	D_2_ (NSFO)	40.45 ± 1.69	42.46 ± 1.43	42.68 ± 1.88
	D_3_ (NSEO)	39.08 ± 1.49	41.79 ± 2.03	41.82 ± 1.86

LDL (mg/dL)	D_1_ (control)	47.71 ± 2.75^d^	72.79 ± 2.36^b^	83.41 ± 4.23^a^
	D_2_ (NSFO)	51.13 ± 2.14^c^	48.54 ± 1.63^d^	38.99 ± 1.72^e^
	D_3_ (NSEO)	57.44 ± 2.19^c^	53.20 ± 2.59^c^	46.02 ± 2.04^d^

Triglycerides (mg/dL)	D_1_ (control)	102.45 ± 5.91^b^	103.96 ± 3.37^b^	130.34 ± 6.61^a^
	D_2_ (NSFO)	93.89 ± 3.93^d^	89.50 ± 3.01^e^	86.48 ± 3.81^e^
	D_3_ (NSEO)	98.94 ± 3.77^c^	94.64 ± 4.61^d^	93.67 ± 4.16^d^

Means sharing same letters in a column/row do not differ significantly at *P* < 0.05.

**Table 4:** Indices of antioxidants damage in diabetic rats treated with NSFO and NSEO.

Parameters	Diets	Study intervals (days)		
		0	28	56
Total antioxidants capacity (TAC) (IU/mL)	D_1_ (control)	0.52 ± 0.03^de^	0.44 ± 0.01^e^	0.29 ± 0.02^f^
	D_2_ (NSFO)	0.56 ± 0.02^d^	0.65 ± 0.02^c^	0.78 ± 0.03^b^
	D_3_ (NSEO)	0.47 ± 0.02^de^	0.67 ± 0.03^c^	0.91 ± 0.04^a^

MDA (nmol/g)	D_1_ (control)	6.61 ± 0.38^c^	9.02 ± 0.29^b^	10.51 ± 0.53^a^
	D_2_ (NSFO)	7.00 ± 0.29^c^	5.86 ± 0.20^d^	4.89 ± 0.21^e^
	D_3_ (NSEO)	7.06 ± 0.27^c^	3.66 ± 0.18^f^	3.28 ± 0.15^f^

Conjugated dienes (CD) (nmol/g)	D_1_ (control)	2.25 ± 0.13^c^	2.63 ± 0.08^b^	3.01 ± 0.15^a^
	D_2_ (NSFO)	2.08 ± 0.09^c^	1.90 ± 0.06^cd^	1.84 ± 0.08^d^
	D_3_ (NSEO)	2.08 ± 0.08^c^	1.58 ± 0.08^e^	1.41 ± 0.061^e^

Means sharing same letters in a column/row do not differ significantly at *P* < 0.05.

**Table 5:** Correlation matrix of some important parameters in diabetic rats.

	Chl	HDL	TG	LDL	Gl.	Insulin	MDA	TAC	CD
Chl	1.00								
HDL	−0.82**	1.00							
TG	0.94**	−0.80*	1.00						
LDL	0.99**	−0.87**	0.90**	1.00					
Gl	0.93**	−0.75*	0.89**	0.92**	1.00				
Insulin	−0.57^ns^	0.27^ns^	−0.65^ns^	−0.50^ns^	−0.74*	1.00			
MDA	0.74*	−0.69^ns^	0.78*	0.73*	0.86**	−0.72*	1.00		
TAC	−0.68^ns^	0.66^ns^	−0.70*	−0.68^ns^	−0.77*	0.69^ns^	−0.96**	1.00	
CD	0.86**	−0.72*	0.87**	0.83**	0.96**	−0.79**	0.94**	−0.86**	1.00

*Significant (*P* < 0.05); **highly significant (*P* < 0.01); and ns: non-significant.

Chl: cholesterol; HDL: high density lipoprotein; TG: triglycerides; LDL: low density lipoprotein; Gl: glucose; MDA: malonaldehyde; TAC: total antioxidant capacity; CD: conjugated dienes.
